# Case report: Pharmacokinetic interaction involving sirolimus and regorafenib in patients with post-transplant recurrent hepatocellular carcinoma

**DOI:** 10.3389/fphar.2025.1472896

**Published:** 2025-02-19

**Authors:** Kongcai Zhu, Fang Xiong, Haihong Bai, Wei Liu

**Affiliations:** ^1^ Department of Pharmacy, Beijing YouAn Hospital, Capital Medical University, Beijing, China; ^2^ Department of Oncology, Beijing YouAn Hospital, Capital Medical University, Beijing, China

**Keywords:** regorafenib, sirolimus trough concentration, drug interaction, case report, hepatocelluar carcinoma, liver transplantation

## Abstract

**Background:**

Sirolimus is primarily metabolized by CYP3A4 and transported by P-gp. Drug interactions that affect this pathway can alter its plasma exposures, resulting in untargeted sirolimus concentrations.

**Case summary:**

In this case report, we investigate a pharmacokinetic drug-drug interaction between regorafenib and sirolimus, mediated by CYP3A4 and P-gp, in a 56-year-old Chinese male with recurrent hepatocellular carcinoma post-liver transplantation. In this case, the patient’s baseline sirolimus trough blood concentration was 5.0 ng/mL prior to initiating a new cycle of regorafenib (80 mg once daily). Following a 7-day administration period of regorafenib, a notable elevation in sirolimus trough blood concentration to 12.3 ng/mL was observed. Upon cessation of regorafenib therapy for one week, the sirolimus trough blood concentration reverted back to 5.2 ng/mL. Nevertheless, upon resumption of regorafenib (160 mg once daily) treatment for an additional 10 days, the sirolimus trough blood concentration experienced a recurrence of increase, reaching 11.0 ng/mL. Following the confirmation of tumor progression, the discontinuation of regorafenib was deemed necessary. Consequently, a subsequent medical evaluation of the patient’s sirolimus trough blood concentration, undertaken precisely one month after cessation of regorafenib therapy, revealed a concentration level of 2.8 ng/mL. Based on the Drug Interaction Probability Scale, this interaction was deemed probable.

**Conclusion:**

Regorafenib exerts a regulatory influence on the blood concentrations of sirolimus by inhibiting the activity of CYP3A4 and P-gp, potentially altering its pharmacokinetic profile. Given the potential for both excessive and inadequate immunosuppression to adversely affect patients with recurrent hepatocellular carcinoma post-liver transplantation, clinicians must maintain a heightened awareness of this drug-drug interaction.

## Introduction

Regorafenib is an oral multi-kinase inhibitor that targets multiple protein kinases, including those implicated in tumor angiogenesis, tumorigenesis, and metastasis (European Medicines Agency, 2024b; Australian Public Assessment Report for Regorafenib, 2014). It has been approved for the treatment of hepatocellular carcinoma (HCC) in the United States, the European Union, and China due to its significant efficacy and favorable safety profile. *In vitro* studies have demonstrated that regorafenib exhibits inhibitory effects on CYP3A4, P-glycoprotein (P-gp), and Breast Cancer Resistance Protein (BCRP) (European Medicines Agency, 2024b; Australian Public Assessment Report for Regorafenib, 2014). Consequently, increased vigilance is warranted in monitoring the interactions of regorafenib with other drugs, particularly during the metabolic phase, as patients with recurrent HCC post-liver transplantation (LT) are more prone to undergo complex combination therapy.

After LT for HCC patient, an immunosuppressive therapy is also crucial to prevent rejection reactions and enhance survival. Sirolimus, a mammalian target of rapamycin (mTOR) inhibitor, is currently primarily used in recipients with renal dysfunction post-LT, intolerable adverse reactions to calcineurin inhibitors (CNIs), and a risk of tumor recurrence. It can ameliorate renal dysfunction induced by CNIs, alleviate adverse reactions to CNIs, and exhibit antitumor effects (Shenoy et al., 2007; Harper et al., 2011; Xu et al., 2019). The target whole blood trough concentration of sirolimus is 5 ng/mL according to the practice guideline of the American Association for the Study of Liver Diseases and the American Society of Transplantation (Lucey et al., 2013), whereas in the Chinese expert consensus on clinical application of inhibitors of mammalian target of rapamycin in liver transplant recipients (Feng et al., 2023), it ranges from 6 to 10 ng/mL. *In vivo*, sirolimus is extensively metabolized by CYP3A4 in the intestinal wall and liver. Additionally, sirolimus is a substrate for the multidrug efflux pump, P-gp, located in the small intestine (European Medicines Agency, 2024c). Drugs that influence the levels or activity of CYP3A4 or P-gp are expected to alter the pharmacokinetics of sirolimus. Given the metabolism of regorafenib and sirolimus, it cannot be ruled out that their simultaneous use increases the risk of clinically significant drug-drug interactions.

The recurrence rates of HCC after LT are 10%–20% in the first year following the procedure (To et al., 2024). Regorafenib has been shown to enhance overall survival (OS) in patients with post-transplant recurrent HCC who have progressed following sorafenib therapy (Iavarone et al., 2019). Moreover, considering the need for long-term immunosuppressive therapy after LT, concurrent use of regorafenib and sirolimus is common in this patient cohort. To the best of our knowledge, there are currently no published studies investigating the potential drug-drug interaction between regorafenib and sirolimus. This case report is the first to analyze CYP3A4 and P-gp-mediated drug-drug interactions between regorafenib and sirolimus in patients with recurrent HCC post-LT. Our study revealed that regorafenib can increase the whole blood trough concentration of sirolimus by over 100% *in vivo*.

## Case description

A 56-year-old Chinese male patient with a decade-long history of fatty liver disease underwent LT for HCC on 19 October 2022. In May 2023, an elevated alpha-fetoprotein (AFP) level was detected. Consequently, he commenced treatment with lenvatinib capsules and underwent radiofrequency ablation therapy in November 2023. Due to a lack of improvement in his condition, the patient began taking regorafenib, one tablet daily, in January 2024. In March 2024, the patient was admitted to Beijing You’an Hospital. MRI enhanced scan imaging revealed multiple new lesions in the liver, small, enhanced nodules in the abdominal cavity and anterior abdominal wall, suggesting possible metastasis. The patient’s AFP was 2,895.5 ng/mL. Upon admission, the patient’s anti-rejection regimen consisted of sirolimus tablets administered at a dose of 1 mg once daily, along with mycophenolate mofetil capsules at a dose of 250 mg twice daily.

## Antineoplastic therapy and sirolimus whole blood trough concentration

At the time of admission, the patient had been off regorafenib for 1 week. On the morning of March 12th, the trough blood concentration of sirolimus in the patient was measured using liquid chromatography-mass spectrometry (LC-MS), yielding a value of 5.0 ng/mL. The patient then initiated a new treatment cycle with regorafenib on March 12th at a dose of 80 mg daily. On March 13th, the patient underwent transarterial chemoembolization (TACE) therapy. Subsequently, on March 19th, when the blood concentration of mycophenolic acid was being measured, the trough blood concentration of sirolimus was also reassessed, yielding a value of 12.3 ng/mL, approximately double the previous measurement. Regarding the elevated blood concentration of sirolimus, we suspect it may be correlated with the administration of regorafenib, or potentially due to fluctuations in liver function following TACE. On 15 April 2024, the patient was re-admitted to Beijing You’an Hospital for further anti-tumor treatment. Notably, the patient had discontinued regorafenib exactly 1 week prior to admission and required the initiation of a new treatment cycle with regorafenib. Given that the second measurement of sirolimus blood concentration was significantly higher than the initial measurement during the patient’s previous hospitalization, we repeated the sirolimus trough blood concentration test on the morning of April 16th, yielding a result of 5.2 ng/mL. The patient subsequently commenced a new round of regorafenib treatment on April 16th, administered at a dosage of 120 mg once daily. The patient’s AFP was 10,020.3 ng/mL. Due to suspicions that the fluctuations in sirolimus blood concentration observed during the previous hospitalization might be linked to regorafenib intake, we re-assessed the sirolimus trough blood concentration on April 26th. The test result indicated a concentration of 11.0 ng/mL. MRI imaging conducted following the patient’s admission indicated tumor progression. Consequently, regorafenib was discontinued on April 26th, and ramucirumab was initiated as the subsequent treatment modality. On June 7th, a repeat measurement of the patient’s sirolimus trough blood concentration revealed a value of 2.8 ng/mL, falling below the target therapeutic concentration, without any alteration to the sirolimus dosage prescribed in the previous regimen. On June 14th, the patient developed progressive hepatic dysfunction. The absence of a liver biopsy made it impossible to definitively determine the underlying cause of the observed liver abnormalities. The patient’s laboratory test results were presented in Table 1.

**TABLE 1:** Summary of the patient’s laboratory test outcomes.

	7 days after the suspension of regorafenib (March 12th)	7 days after the intro-duction of regorafenib (March 19th)	7 days after the suspension of regorafenib (April 16th)	10 days after the intro-duction of regorafenib (April 26th)	One month after the dis-continuation of regorafenib (June 7th)
ALT (U/L)	20	44	21	20	29
AST (U/L)	38	37	30	55	67
TBil (μmol/L)	3.4	9.2	5.3	6.0	3.9
DBil (μmol/L)	1.5	4.1	2.8	2.9	1.6
γ-GGT (U/L)	38	84	117	62	109
ALP (U/L)	98	145	134	124	138
CRE (μmol/L)	157	148	171	153	186
ALB (g/L)	33.1	33	36.4	33.3	34.9
HGB (g/L)	95	90	98	98	102
PLT (*10^9/L)	112	141	144	117	131
WBC (*10^9/L)	4.81	5.92	6.34	7.71	3.57
PT (s)	8.4	9.9	9.5	8.9	8.3

ALT, alanine aminotransferase; AST, aspartate aminotransferase; TBil, total bilirubin; DBil, direct bilirubin; γ-GGT, γ-glutamyltranspeptidase; ALP, alkaline phosphatase; CRE, creatinine; ALB, albumin; Cmin^ss^, trough steady-state concentrations; HGB, hemoglobin; PLT, platelet; WBC, white blood cell; PT, prothrombin time.

## Discussion

### Fluctuations of sirolimus trough blood concentration associated with regorafenib

**FIGURE 1:**
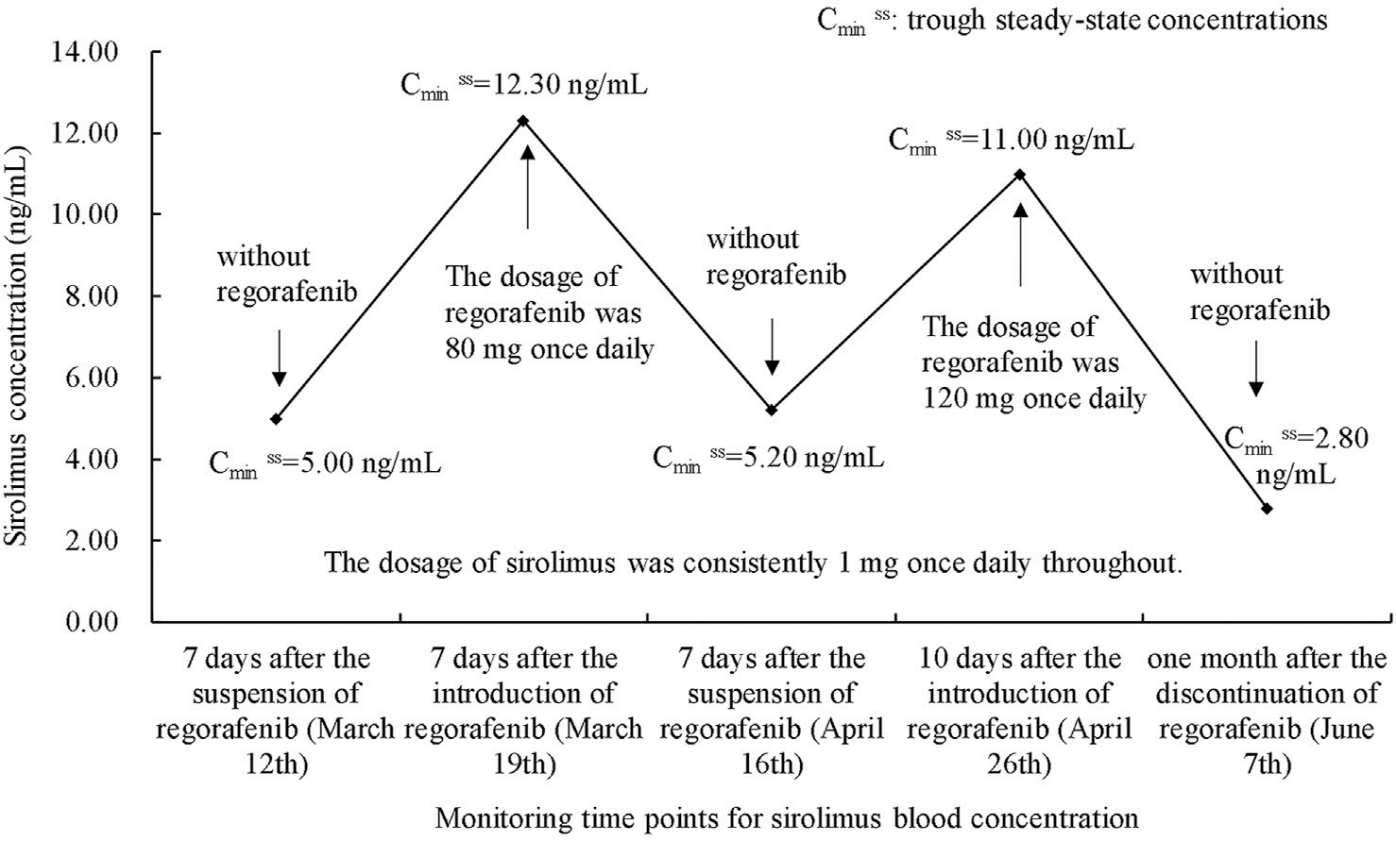
Follow-up of sirolimus trough blood concentrations before and after regorafenib introduction.

To the best of our knowledge, we hereby introduce the first report documenting a pharmacokinetic drug-drug interaction between regorafenib and sirolimus. Based on the Drug Interaction Probability Scale (DIPS) (Horn et al., 2007), the interaction was deemed probable, with a DIPS score of 6. In this case, where no modifications were made to other concomitantly administered treatments, the introduction of regorafenib resulted in a 146% increase in the plasma concentration of sirolimus. Nevertheless, upon fully discontinuing regorafenib for a period of 1 month, the trough concentration of sirolimus subsequently fell below the desired target concentration. The variations in sirolimus trough blood concentrations were depicted in Figure 1. During our initial monitoring, we observed fluctuations in the patient’s sirolimus blood concentration that coincided with the timing of the TACE treatment, but it remained unclear whether the TACE was the primary cause of the sirolimus blood concentration fluctuations. Upon the patient’s second admission, we reassessed sirolimus levels. Notably, during this hospitalization, the patient did not undergo TACE, and liver function remained stable. Despite this, sirolimus fluctuations persisted. Based on these findings, we concluded that drug interactions were the primary cause of the variability. However, it is important to note that during the second admission, the dose of regorafenib was increased compared to the first, yet the extent of sirolimus fluctuations decreased from 146% to 112%. This suggests that while TACE surgery does affect sirolimus levels, it is not the primary factor driving significant fluctuations in this case. Notably, throughout the entire treatment course, the dosing regimen for sirolimus and mycophenolate mofetil remained unchanged.

### Potential underlying mechanism of regorafenib affecting the blood concentration of sirolimus

*In vitro* studies have established that regorafenib is primarily metabolized via cytochrome CYP3A4 and UGT1A9 pathways, exhibiting an inhibitory influence on P-gp (IC_50_ 2.2 µM), BCRP (IC_50_ 56 nM), and CYP3A4 (IC_50_ 5.8/8.3/9.1/10.4 µM, Ki 11.1 µM) at clinically relevant concentrations of total drug (about 7.2 µM) (European Medicines Agency, 2024b; Australian Public Assessment Report for Regorafenib, 2014). Notably, a study conducted by Iavarone M et al. (Iavarone et al., 2019) necessitated the adjustment of immunosuppressive medications in five patients who presented with elevated plasma concentrations of everolimus and tacrolimus subsequent to 15 days of regorafenib therapy. The investigators suspected that this elevation stemmed from regorafenib’s inhibition of CYP3A4 activity, given that both tacrolimus and everolimus are CYP3A4 substrates. Furthermore, as tacrolimus and everolimus are also substrates for P-gp (European Medicines Agency, 2024a; Saeki et al., 1993), it is reasonable to hypothesize that the inhibitory action of regorafenib on P-gp may contribute to the observed elevation in their blood concentrations. Similarly, sirolimus, reported herein, is a substrate for both CYP3A4 and P-gp (European Medicines Agency, 2024c), resulting in an observed drug interaction comparable to that with tacrolimus and everolimus. However, D. Strumberg et al. reported that regorafenib did not significantly elevate the exposure levels of digoxin, a known substrate of P-gp (Strumberg et al., 2016). However, a notable limitation in their study is the fact that digoxin did not attain a pharmacokinetic steady state, thereby hindering the ability to draw definitive conclusions. Consequently, the precise mechanism underpinning the drug-drug interaction between regorafenib and sirolimus necessitates further *in vitro* investigations for verification.

### Adverse clinical consequences of excessively high or low blood concentrations of sirolimus

In our case report, a marked elevation of 146% in the *in vivo* blood concentrations of sirolimus was observed subsequent to the administration of regorafenib. Kahan’s (Kahan et al., 2000) research indicates that the trough concentration of sirolimus is correlated with adverse reactions. As the trough concentration of sirolimus escalates, there is a consequential escalation in the prevalence of hypertriglyceridemia (featuring an inflection point at 11 ng/mL), thrombocytopenia (manifesting an inflection point at 14 ng/mL), and leukopenia (similarly, with an inflection point at 15 ng/mL), indicating a definitive relationship between drug concentration and these adverse effects. Furthermore, for LT recipients with recurrent HCC, the Chinese Clinical Practice Guidelines on LT for HCC advocate for individualized low-dose immunosuppressive protocols, as high-dose immunosuppressant therapy disrupts immune homeostasis, thereby fostering tumor recurrence and metastasis (Xu et al., 2019). In the context of LT for patients with HCC, the likelihood of tumor recurrence is intricately tied to the tumor’s aggressiveness and the recipient’s immune competence. During periods of profound immunosuppression, the delicate balance of the immune surveillance system is disrupted, fostering an environment conducive to tumor recurrence and metastasis. A retrospective study conducted by Vivarelli et al. has demonstrated that elevated blood concentrations of CNI, namely, tacrolimus exceeding a trough level of 10 ng/mL or cyclosporine A exceeding a trough level of 220 ng/mL, markedly augment the risk of tumor recurrence by a magnitude of 5–6 times subsequent to LT for HCC (Vivarelli et al., 2008). However, Li’s study (Li et al., 2020) reported a successful case of managing diffuse bilateral lung metastasis in a post-LT patient with giant HCC that exceeded transplant criteria, using combination therapy with regorafenib and sirolimus. Notably, the study did not include details on sirolimus blood concentrations. In contrast, our patient experienced rapid tumor progression despite regorafenib treatment. Therefore, the potential impact of elevated sirolimus levels on therapeutic outcomes in LT patients with recurrent HCC requires further investigation.

Moreover, subsequent to the one-month discontinuation of regorafenib administration, we observed a significant decline in the trough plasma concentration of sirolimus, falling outside the clinically established therapeutic target range. Kahan’s (Kahan et al., 2000) research indicates a significant association between sirolimus trough blood levels, expressed either as observed values (<5 ng/mL) or as dose-corrected parameters (<1.7 ng/mL per mg of administered drug), and the occurrence and severity of acute rejection episodes. In the subsequent therapeutic phase, the patient exhibited progressive deterioration of hepatic function. Nevertheless, the absence of a liver biopsy hindered the ability to conclusively discern whether the observed hepatic abnormalities were due to tumor progression or indicative of an organ rejection process.

Therefore, our case report emphasizes the importance of vigilant monitoring of sirolimus trough blood concentrations in LT patients with recurrent HCC undergoing concurrent sirolimus and regorafenib therapy, enabling proactive management of drug toxicity and efficacy, as well as the refinement of personalized, low-dose immunosuppressive regimens.

## Conclusion

In conclusion, this case report underscores the significance of acknowledging the potential pharmacokinetic interaction involving CYP3A4 and P-gp inhibition by regorafenib in LT recipients with recurrent HCC undergoing treatment with regorafenib and sirolimus. Given this context, the adoption of meticulous management strategies and individualized therapeutic drug monitoring is imperative to ensure the safe navigation of these potential drug-drug interactions. Consequently, current evidence underscores the need for rigorous monitoring of sirolimus blood levels in LT patients with recurrent HCC undergoing regorafenib therapy, with subsequent adjustment of sirolimus dosing as warranted.

## Data Availability

The original contributions presented in the study are included in the article/supplementary material, further inquiries can be directed to the corresponding author.
